# The Role of Immunohistochemistry Studies in Distinguishing Malignant Mesothelioma from Metastatic Lung Carcinoma in Malignant Pleural Effusion

**DOI:** 10.30699/IJP.14.2.122

**Published:** 2019-01-10

**Authors:** Monireh Halimi, Samad BeheshtiRouy, Davood Salehi, Seyed Ziaeddin Rasihashemi

**Affiliations:** 1 *Department of Pathology, Tabriz University of Medical Sciences, Tabriz, Iran*; 2 *Department of Cardiothoracic Surgery, Imam Reza Hospital, Tabriz University of Medical Sciences, Tabriz, Iran*

**Keywords:** Mesothelioma, Immunohistochemistry, Marker, Lung Adenocarcinoma

## Abstract

**Background and Objective:**

Early diagnosis of malignant pleural mesothelioma (MPM) is the key point of its treatment. The main problem is the precise diagnosis of mesothelioma and its differentiation from metastatic lung adenocarcinoma. Mesothelioma exhibits complex immunohistochemical characteristics. The aim of this study was to study hybrid immunohistochemistry in the differential diagnosis of primary malignant pleural effusion from metastatic pulmonary cancers.

**Material and Methods:**

Twenty tissue samples in paraffin blocks from the pathology department of Imam Reza Hospital in Tabriz whose pathology reports cited mesothelioma or metastatic lung adenocarcinomas, were included in the studies. These tissues were deemed appropriate for IHC in terms of tissue quality and quantity. They were studied and evaluated for pathological markers.

**Results:**

In patients with adenocarcinoma CK7 in 100% of patients (13 patients), TTF1 in 61.5% of patients (8 patients) and CEA in 53.8% of patients (7 patients) were positive, but HBME1 and Calretinin were negative for all patients. In patients with mesothelioma, HBME1 and Calretinin were positive in 100% of patients (7 patients) and TTF1, CEA and CK7 were negative.

**Conclusion:**

The results of this study showed that CEA, CK7, TTF1, Calretinin and HBME1 are suitable criteria for differentiating between metastatic lung adenocarcinoma and mesothelioma, and can differentiate the mesothelioma and adenocarcinoma with high accuracy.

## Introduction

MPM is an uncommon cancer with a poor prognosis and very limited treatment options. MPM originates from mesothelial cells (1). Contrary to lung cancer, cigarette smoking is not a predisposing factor for this cancer, but long-term exposure to asbestos is one of its major causes. The key point is the early diagnosis and aggressive treatment of the disease (2). 

The main symptoms are dyspnea, chest pain and fluid accumulation in the pleural cavity (3). The most common radiologic manifestations are pleural effusion, pleural cavity and pleural thickening. The diagnosis of MPM is usually made by way of cutaneous needle biopsy, thoracoscopic biopsy or open pleural biopsy. Based on cytologic analysis of the pleural fluid, the diagnosis and differentiation of MPM from metastatic carcinoma is difficult or impossible (4, 5). Cancers other than pulmonary cancers may also exhibit pleural metastases. To differentiate between MPM and metastatic pulmonary pleural effusion, a histopathologic analysis is required (6). Based on histopathologic characteristics, immunohistochemistry (IHC) can provide valuable information for the diagnosis and categorization of different tumors and their subtypes and the source of metastatic tumors, thereby aiding in the determination of the suitable treatment protocols (7). In the last decade, a wide range of antibodies or IHC markers have been developed and used for this differentiation (8).

 Given that no studies have been done in this regard in our country, and considering the fact that there are many disparate studies in this field , so in this study, we decided to evaluate the pathologic value of CEA, CK7, TTF1, Calretinin and HBME1 in differentiating mesothelioma and adeno-carcinoma .

## Materials and Methods

In this descriptive-analytical study, 20 samples were extracted from the pathology department of Imam Reza hospital in Tabriz, between 2009 and 2013 that were reported as MPM or metastatic lung adenocarcinomas, and whose tissue samples in the paraffin block were suitable for immune-histochemistry analysis in terms of tissue quality and quantity. The only samples that were included in the study were the ones that had pathological data confirming the diagnosis of MPM or metastatic adenocarcinomas of the lung as indicated in previous reports. After selecting the samples, information about the disease (tumor size, stage and microscopic degree of the tumor) and the patients (age and sex) were extracted from the clinical and pathological records and recorded in the information form. The pathologist did not have access to this information and the study was single-blinded. 

At first, microscopic slides containing 5 micron sections of the paraffin blocks from the samples were prepared and stained with Hematoxylin-Eosin. Another expert pathologist had reported these samples to exclude any misdiagnosis. After confirmation of the first diagnosis, the samples were prepared for immunohistochemistry staining (TTF1-CK7-HBME1-Calretinin-CEA) in a standard method.

The immunohistochemistry study of the samples was performed on 3 μm paraffin-stained tissue (IHC) sections in formalin on glass slides using antibodies (TTF1-CK7-HBME1-Calretinin-CEA). All sections were initially dewaxed, de-humidified and placed in a microwave in a fresh Citrate HCl buffer solution with pH = 6 for 10 min. Then, they were washed at room temperature in phosphate buffered saline (PBS) and incubated with anti-Ki67 antigen (DAKO Antibodies) at 0.1 dilution for 1 hour. By cooling the slices in hydrogen peroxide at room temperature, peroxide activity was blocked from the tissue. Samples that had very little or no tumor cells were excluded from the study.

The sections were washed with PBS and incubated with biotinylated antibodies for 30 minutes, followed again by washing with PBS and incubation with Peroxidase Labeled Streptavidin for 30 minutes. In the next step after washing the samples again with PBS, they were covered with chromogen 3.3 Diaminobenzidine Hydro Chloride (DAB). The sections were then stained with Ethyl-Green and re-humidified and finally covered with lamellas. Meanwhile, in order to control the quality of work, a positive control and a negative control were taken alongside each of the sections. Tumor margin samples were selected as negative controls. It should be noted that the types of tumor margins were collected from distant areas of the tumor that apparently lacked tumor involvement and were removed from the lamella in the negative control of the first antibody.

Cell counting was performed on 100 cells in the 10 regions with the highest dye content, and the percentage of positive Labeling Index cells was recorded. For an image count of the brown-colored cells seen in the nucleus, the Image Focus Capture Version 2.5.2006 software and the BX43 microscope (Olympus made in Japan) were used at a magnification of 400, with 10 separate fields with a magnification of 40 in parasol areas. 

The cells were positively evaluated for each antibody, and according to the percentage of colored cells, the samples were divided into two groups: low expression (expressing 48% and less) and high expression (expression of more than 48%) (9). To ensure the accuracy of the test, the experiments were carried out on two phases by two testers. In cases with a disagreement, the samples were re-examined so as to reach a consensus, and in case both anti-infectious agents showed the gene was positive, the samples were considered positive. 

Data was analyzed using the descriptive statistical methods (Mean ± SE), frequency and percentage, and mean difference test for independent groups of quantitative variables and the Chi-square test for qualitative variables. To compare qualitative variables, Fisher’s exact test, Pearson’s Chi-square test, and non-parametric variables were tested using McNemar's test. The independent t-test was used to compare the quantitative variables and analyzed using statistical software 17SPSS ™. In this study, a P-value of less than 0.05 was considered statistically significant.

## Results

In this study, 20 patients were examined, of which 13 patients (9 male and 4 female patients) has adenocarcinomas and 7 patients (3 male and 4 female patients) has mesothelioma. The mean age in patients with adenocarcinoma was 59.53 ± 13.97 years (43-87). In mesothelioma patients, the mean age was 65 ± 15.52 years (38-81). There was no significant difference between the two groups in terms of age (*P* = 0.84). 

HBME1 was positive in all patients with MPM (7 patients) but negative in all patients (13 patients) with adenocarcinoma. This difference was statistically significant and HBME1 was significantly positive in patients with mesothelioma (*P* <0.001).

The current study showed that all cases of MPM had Calretinin as positive marker (7 patients), but it was negative in all patients (13 patients) with adenocarcinoma. There was a statistically significant difference (*P* <0.001). 

 In 8 patients (61.5%) with adenocarcinoma TTF1 was positive, but was negative for all patients (7 patients) with MPM. The difference between the two groups was statistically significant (*P* = 0.015). TTF-1-positive tumors were significant in patients with lung adenocarcinoma.

 Seven patients (53.8%) with adenocarcinomas were positive for CEA, but in all patients (100%) CEA were negative with MPM diagnosis. This difference was statistically significant (*P* = 0.44). CEA was significantly positive in patients with adenocarcinoma. 

CK7 was positive in all patients (100%) with adenocarcinoma, but it was negative in all patients (100%) with MPM. There was a statistically significant difference according to this test (*P* <0.001) and CK7 was significantly positive in patients with adenocarcinoma.

## Discussion

The distinction between MPM and metastatic pleural adenocarcinoma is a diagnostic challenge in patients with malignant pleural effusion. As the incidence of MPM appears to be increasing, it has become a major problem facing surgical pathologists in recent years. However, lung cancer remains one of the most common causes of metastatic pleural effusion. Various pathological techniques have been developed for the separation of MPM and adenocarcinoma, which include histochemical and immunohistochemical analyses (10). 

The morphological differentiation of metastatic adenocarcinoma, malignant epithelial mesothelium and reactive mesothelioma is difficult, therefore additional cytological methods are needed (11). Immunohistochemical staining is now widely used for the cytopathological diagnosis of neoplasms*.* Wide range of antibodies is used to differentiate adenocarcinoma, mesothelioma, and reactive mesothelium cells (12, 13).

Immunohistochemistry provides important indicators for distinguishing between MPM and lung adenocarcinoma (13). Recently, various immunocytochemistry markers (CEA, E-cadherin, Ber-EP4, calretinin, HBME-1, P53, and TTF1) have been used to distinguish between mesothelioma and adenocarcinoma (13). In our study, HBME1 was negative in all patients with adenocarcinoma (100%), but it was positive in all patients (100%) with mesothelioma. In a study by Dino et al., HBME1 was negative in patients with lung adenocarcinoma (14). However, Ordóñez found that HBME1 was positive in 85% of patients with mesothelioma and in 68% of patients with lung adenocarcinoma (15). In a study by Camilla et al., HBME1 has been shown to have high sensitivity for differentiation between lung adenocarcinoma and mesothelioma (16).

 In our study all patients (100%) with adenocarcinoma had negative results for calretinin but it was positive in all patients (100%) with mesothelioma. In the Ordóñez study, Calretinin was positive in 100% of patients with mesothelioma and in 8% of patients with lung adenocarcinoma (15). Camilla and colleagues showed that Calretinin was a high specific mesothelium marker (16). Lucian and colleagues reported that Calretinin had a key role in detecting malignant mesothelioma (18).

 In our study, TTF1 was positive in 8 patients (61.5%) with adenocarcinoma, but negative in all patients (100%) with mesothelioma. In a study by Dino et al., TTF1 was negative in the mesothelioma group but positive in the adenocarcinoma group (14). Ordóñez found that TTF1 was negative in all patients with mesothelioma, but positive in 74% of patients with lung adenocarcinoma (15). Woo et al in a literature review showed that TTF-1 and napsin are the most useful IHC markers for pulmonary adenocarcinoma (19). The results of the NG and colleagues study showed that TTF1 was positive in all patients with lung adenocarcinoma (100% specificity) (20).

 In our study, CEA was positive in 7 patients (53.8%) with adenocarcinoma but negative in all patients (100%) with mesothelioma. Ordóñez also found that CEA was negative in all patients with mesothelioma and positive in 88% of patients with lung adenocarcinoma (15). The results of the Dino et al. study showed that CEA was positive in patients with mesothelioma but was negative in patients with lung adenocarcinoma (14). Carella and colleagues also found that CEA was positive in 85% of patients with adenocarcinoma and in 4% of patients with mesothelioma (13). Su and colleagues showed that CEA has high specificity but low sensitivity in differentiating between adenocarcinoma and mesothelioma (12). 

In our study, CK7 was positive in all patients (100%) with adenocarcinoma, but negative in all patients (100%) with mesothelioma. In a study eighty-six percent (12 of 14) of patients with mesothelioma had positive results for CK7+, but lung adenocarcinomas, both primary and metastatic that were very similar to mesotheliomas regarding expression of CK7. They concluded that adding CK7 to the panel of antibodies is useful in the differential diagnosis of pleural mesothelioma versus metastatic adenocarcinomas because diffuse CK20 positivity seems to be an indicator of metastasis (21).

**Limitations: **Due to very low incidence of mesothelioma, the study sample size was small that may cause some bios. There are different types of Immunohistochemistry markers, therefore finding a specific antibody for mesothelioma needs additional studies.

## Conclusion

Results of our study demonstrated that CEA, CK7, TTF1, Calretinin and HBME1 are proper markers in the differentiation between lung adenocarcinoma and mesothelioma and can be associated with a high precision for mesothelioma and lung adenocarcinoma differentiation.
